# Contesting the “Nature” Of Conformity: What Milgram and Zimbardo's Studies Really Show

**DOI:** 10.1371/journal.pbio.1001426

**Published:** 2012-11-20

**Authors:** S. Alexander Haslam, Stephen. D. Reicher

**Affiliations:** 1 School of Psychology, University of Queensland, St. Lucia, Australia; 2 School of Psychology, University of St. Andrews, St Andrews, Scotland

## Abstract

Understanding of the psychology of tyranny is dominated by classic studies from the 1960s and 1970s: Milgram's research on obedience to authority and Zimbardo's Stanford Prison Experiment. Supporting popular notions of the banality of evil, this research has been taken to show that people conform passively and unthinkingly to both the instructions and the roles that authorities provide, however malevolent these may be. Recently, though, this consensus has been challenged by empirical work informed by social identity theorizing. This suggests that individuals' willingness to follow authorities is conditional on identification with the authority in question and an associated belief that the authority is right.

## Abstract 2

A re-analysis of classic psychology studies suggests that tyranny does not result from blind conformity to rules and roles, but may involve identification with authorities who represent vicious acts as virtuous.

## Introduction

*If men make war in slavish obedience to rules, they will fail.*

Ulysses S. Grant [1]

Conformity is often criticized on grounds of morality. Many, if not all, of the greatest human atrocities have been described as “crimes of obedience” [2]. However, as the victorious American Civil War General and later President Grant makes clear, conformity is equally problematic on grounds of efficacy. Success requires leaders and followers who do not adhere rigidly to a pre-determined script. Rigidity cannot steel them for the challenges of their task or for the creativity of their opponents.

Given these problems, it would seem even more unfortunate if human beings were somehow programmed for conformity. Yet this is a view that has become dominant over the last half-century. Its influence can be traced to two landmark empirical programs led by social psychologists in the 1960s and early 1970s: Milgram's Obedience to Authority research and Zimbardo's Stanford Prison Experiment. These studies have not only had influence in academic spheres. They have spilled over into our general culture and shaped popular understanding, such that “everyone knows” that people inevitably succumb to the demands of authority, however immoral the consequences [3],[4]. As Parker puts it, “the hopeless moral of the [studies'] story is that resistance is futile” [5]. What is more, this work has shaped our understanding not only of conformity but of human nature more broadly [6].

Building on an established body of theorizing in the social identity tradition—which sees group-based influence as meaningful and conditional [7],[8]—we argue, however, that these understandings are mistaken. Moreover, we contend that evidence from the studies themselves (as well as from subsequent research) supports a very different analysis of the psychology of conformity.

## The Classic Studies: Conformity, Obedience, and the Banality Of Evil

In Milgram's work [9],[10] members of the general public (predominantly men) volunteered to take part in a scientific study of memory. They found themselves cast in the role of a “Teacher” with the task of administering shocks of increasing magnitude (from 15 V to 450 V in 15-V increments) to another man (the “Learner”) every time he failed to recall the correct word in a previously learned pair. Unbeknown to the Teacher, the Learner was Milgram's confederate, and the shocks were not real. Moreover, rather than being interested in memory, Milgram was actually interested in seeing how far the men would go in carrying out the task. To his—and everyone else's [11]—shock, the answer was “very far.” In what came to be termed the “baseline” study [12] all participants proved willing to administer shocks of 300 V and 65% went all the way to 450 V. This appeared to provide compelling evidence that normal well-adjusted men would be willing to kill a complete stranger simply because they were ordered to do so by an authority.

Zimbardo's Stanford Prison Experiment took these ideas further by exploring the destructive behaviour of groups of men over an extended period [13],[14]. Students were randomly assigned to be either guards or prisoners within a mock prison that had been constructed in the Stanford Psychology Department. In contrast to Milgram's studies, the objective was to observe the interaction within and between the two groups in the absence of an obviously malevolent authority. Here, again, the results proved shocking. Such was the abuse meted out to the prisoners by the guards that the study had to be terminated after just 6 days. Zimbardo's conclusion from this was even more alarming than Milgram's. People descend into tyranny, he suggested, because they conform unthinkingly to the toxic roles that authorities prescribe without the need for specific orders: brutality was “a ‘natural’ consequence of being in the uniform of a ‘guard’ and asserting the power inherent in that role” [15].

Within psychology, Milgram and Zimbardo helped consolidate a growing “conformity bias” [16] in which the focus on compliance is so strong as to obscure evidence of resistance and disobedience [17]. However their arguments proved particularly potent because they seemed to mesh with real-world examples—particularly evidence of the “banality of evil.” This term was coined in Hannah Arendt's account of the trial of Adolf Eichmann [18], a chief architect of the Nazis' “final solution to the Jewish question” [19]. Despite being responsible for the transportation of millions of people to their death, Arendt suggested that Eichmann was no psychopathic monster. Instead his trial revealed him to be a diligent and efficient bureaucrat—a man more concerned with following orders than with asking deep questions about their morality or consequence.

Much of the power of Milgram and Zimbardo's research derives from the fact that it appears to give empirical substance to this claim that evil is banal [3]. It seems to show that tyranny is a natural and unavoidable consequence of humans' inherent motivation to bend to the wishes of those in authority—whoever they may be and whatever it is that they want us to do. Put slightly differently, it operationalizes an apparent tragedy of the human condition: our desire to be good subjects is stronger than our desire to be subjects who do good.

## Questioning the Consensus: Conformity Isn't Natural and It Doesn't Explain Tyranny

The banality of evil thesis appears to be a truth almost universally acknowledged. Not only is it given prominence in social psychology textbooks [20], but so too it informs the thinking of historians [21],[22], political scientists [23], economists [24], and neuroscientists [25]. Indeed, via a range of social commentators, it has shaped the public consciousness much more broadly [26], and, in this respect, can lay claim to being the most influential data-driven thesis in the whole of psychology [27],[28].

Yet despite the breadth of this consensus, in recent years, we and others have reinterrogated its two principal underpinnings—the archival evidence pertaining to Eichmann and his ilk, and the specifics of Milgram and Zimbardo's empirical demonstrations—in ways that tell a very different story [29].

First, a series of thoroughgoing historical examinations have challenged the idea that Nazi bureaucrats were ever simply following orders [19],[26],[30]. This may have been the defense they relied upon when seeking to minimize their culpability [31], but evidence suggests that functionaries like Eichmann had a very good understanding of what they were doing and took pride in the energy and application that they brought to their work. Typically too, roles and orders were vague, and hence for those who wanted to advance the Nazi cause (and not all did), creativity and imagination were required in order to work towards the regime's assumed goals and to overcome the challenges associated with any given task [32]. Emblematic of this, the practical details of “the final solution” were not handed down from on high, but had to be elaborated by Eichmann himself. He then felt compelled to confront and disobey his superiors—most particularly Himmler—when he believed that they were not sufficiently faithful to eliminationist Nazi principles [19].

Second, much the same analysis can be used to account for behavior in the Stanford Prison Experiment. So while it may be true that Zimbardo gave his guards no direct orders, he certainly gave them a general sense of how he expected them to behave [33]. During the orientation session he told them, amongst other things, “You can create in the prisoners feelings of boredom, a sense of fear to some degree, you can create a notion of arbitrariness that their life is totally controlled by us, by the system, you, me… We're going to take away their individuality in various ways. In general what all this leads to is a sense of powerlessness” [34]. This contradicts Zimbardo's assertion that “behavioral scripts associated with the oppositional roles of prisoner and guard [were] the sole source of guidance” [35] and leads us to question the claim that conformity to these role-related scripts was the primary cause of guard brutality.

But even with such guidance, not all guards acted brutally. And those who did used ingenuity and initiative in responding to Zimbardo's brief. Accordingly, after the experiment was over, one prisoner confronted his chief tormentor with the observation that “If I had been a guard I don't think it would have been such a masterpiece” [34]. Contrary to the banality of evil thesis, the Zimbardo-inspired tyranny was made possible by the active engagement of enthusiasts rather than the leaden conformity of automatons.

Turning, third, to the specifics of Milgram's studies, the first point to note is that the primary dependent measure (flicking a switch) offers few opportunities for creativity in carrying out the task. Nevertheless, several of Milgram's findings typically escape standard reviews in which the paradigm is portrayed as only yielding up evidence of obedience. Initially, it is clear that the “baseline study” is not especially typical of the 30 or so variants of the paradigm that Milgram conducted. Here the percentage of participants going to 450 V varied from 0% to nearly 100%, but across the studies as a whole, a majority of participants chose not to go this far [10],[36],[37].

Furthermore, close analysis of the experimental sessions shows that participants are attentive to the demands made on them by the Learner as well as the Experimenter [38]. They are torn between two voices confronting them with irreconcilable moral imperatives, and the fact that they have to choose between them is a source of considerable anguish. They sweat, they laugh, they try to talk and argue their way out of the situation. But the experimental set-up does not allow them to do so. Ultimately, they tend to go along with the Experimenter if he justifies their actions in terms of the scientific benefits of the study (as he does with the prod “The experiment requires that you continue”) [39]. But if he gives them a direct order (“You have no other choice, you must go on”) participants typically refuse. Once again, received wisdom proves questionable. The Milgram studies seem to be less about people blindly conforming to orders than about getting people to believe in the importance of what they are doing [40].

## Tyranny as a Product of Identification-Based Followership

Our suspicions about the plausibility of the banality of evil thesis and its various empirical substrates were first raised through our work on the BBC Prison Study (BPS [41]). Like the Stanford study, this study randomly assigned men to groups as guards and prisoners and examined their behaviour with a specially created “prison.” Unlike Zimbardo, however, we took no leadership role in the study. Without this, would participants conform to a hierarchical script or resist it?

The study generated three clear findings. First, participants did not conform automatically to their assigned role. Second, they only acted in terms of group membership to the extent that they actively identified with the group (such that they took on a social identification) [42]. Third, group identity did not mean that people simply accepted their assigned position; instead, it empowered them to resist it. Early in the study, the Prisoners' identification as a group allowed them successfully to challenge the authority of the Guards and create a more egalitarian system. Later on, though, a highly committed group emerged out of dissatisfaction with this system and conspired to create a new hierarchy that was far more draconian.

Ultimately, then, the BBC Prison Study came close to recreating the tyranny of the Stanford Prison Experiment. However it was neither passive conformity to roles nor blind obedience to rules that brought the study to this point. On the contrary, it was only when they had internalized roles and rules as aspects of a system with which they identified that participants used them as a guide to action. Moreover, on the basis of this shared identification, the hallmark of the tyrannical regime was not conformity but creative leadership and engaged followership within a group of true believers (see also [43],[44]). As we have seen, this analysis mirrors recent conclusions about the Nazi tyranny. To complete the argument, we suggest that it is also applicable to Milgram's paradigm.

The evidence, noted above, about the efficacy of different “prods” already points to the fact that compliance is bound up with a sense of commitment to the experiment and the experimenter over and above commitment to the learner (S. Haslam, SD Reicher, M. Birney, unpublished data) [39]. This use of prods is but one aspect of Milgram's careful management of the paradigm [13] that is aimed at securing participants' identification with the scientific enterprise.

Significantly, though, the degree of identification is not constant across all variants of the study. For instance, when the study is conducted in commercial premises as opposed to prestigious Yale University labs one might expect the identification to diminish and (as our argument implies) compliance to decrease. It does. More systematically, we have examined variations in participants' identification with the Experimenter and the science that he represents as opposed to their identification with the Learner and the general community. They always identify with both to some degree—hence the drama and the tension of the paradigm. But the degree matters, and greater identification with the Experimenter is highly predictive of a greater willingness among Milgram's participants to administer the maximum shock across the paradigm's many variants [37].

However, some of the most compelling evidence that participants' administration of shocks results from their identification with Milgram's scientific goals comes from what happened after the study had ended. In his debriefing, Milgram praised participants for their commitment to the advancement of science, especially as it had come at the cost of personal discomfort. This inoculated them against doubts concerning their own punitive actions, but it also it led them to support more of such actions in the future. “I am happy to have been of service,” one typical participant responded, “Continue your experiments by all means as long as good can come of them. In this crazy mixed up world of ours, every bit of goodness is needed” (S. Haslam, SD Reicher, K Millward, R MacDonald, unpublished data).

## Conclusion

The banality of evil thesis shocks us by claiming that decent people can be transformed into oppressors as a result of their “natural” conformity to the roles and rules handed down by authorities. More particularly, the inclination to conform is thought to suppress oppressors' ability to engage intellectually with the fact that what they are doing is wrong.

Although it remains highly influential, this thesis loses credibility under close empirical scrutiny. On the one hand, it ignores copious evidence of resistance even in studies held up as demonstrating that conformity is inevitable [17]. On the other hand, it ignores the evidence that those who do heed authority in doing evil do so knowingly not blindly, actively not passively, creatively not automatically. They do so out of belief not by nature, out of choice not by necessity. In short, they should be seen—and judged—as engaged followers not as blind conformists [45].

What was truly frightening about Eichmann was not that he was unaware of what he was doing, but rather that he knew what he was doing and believed it to be right. Indeed, his one regret, expressed prior to his trial, was that he had not killed more Jews [19]. Equally, what is shocking about Milgram's experiments is that rather than being distressed by their actions [46], participants could be led to construe them as “service” in the cause of “goodness.”

To understand tyranny, then, we need to transcend the prevailing orthodoxy that this derives from something for which humans have a natural inclination—a “Lucifer effect” to which they succumb thoughtlessly and helplessly (and for which, therefore, they cannot be held accountable). Instead, we need to understand two sets of inter-related processes: those by which authorities advocate oppression of others and those that lead followers to identify with these authorities. How did Milgram and Zimbardo justify the harmful acts they required of their participants and why did participants identify with them—some more than others?

These questions are complex and full answers fall beyond the scope of this essay. Yet, regarding advocacy, it is striking how destructive acts were presented as constructive, particularly in Milgram's case, where scientific progress was the warrant for abuse. Regarding identification, this reflects several elements: the personal histories of individuals that render some group memberships more plausible than others as a source of self-definition; the relationship between the identities on offer in the immediate context and other identities that are held and valued in other contexts; and the structure of the local context that makes certain ways of orienting oneself to the social world seem more “fitting” than others [41],[47],[48].

At root, the fundamental point is that tyranny does not flourish because perpetrators are helpless and ignorant of their actions. It flourishes because they actively identify with those who promote vicious acts as virtuous [49]. It is this conviction that steels participants to do their dirty work and that makes them work energetically and creatively to ensure its success. Moreover, this work is something for which they actively wish to be held accountable—so long as it secures the approbation of those in power.
